# Targeting the Secretin Receptor in Macrophages Attenuates Silica‐Induced Pulmonary Fibrosis

**DOI:** 10.1111/cpr.70131

**Published:** 2025-09-18

**Authors:** Yaqian Li, Tian Li, Fuyu Jin, Shupeng Liu, Dingjie Xu, Zhongqiu Wei, Xuemin Gao, Wenchen Cai, Na Mao, Fang Yang, Haibo Zhang, Yiwei Shi, Hong Xu

**Affiliations:** ^1^ College of TCM, North China University of Science and Technology Tangshan China; ^2^ College of Nursing, North China University of Science and Technology Tangshan China; ^3^ School of Public Health, North China University of Science and Technology Tangshan China; ^4^ Basic Medicine College North China University of Science and Technology Tangshan China; ^5^ Department of Anesthesiology and Pain Medicine, Department of Physiology, Interdepartmental Division of Critical Care Medicine University of Toronto Toronto Ontario Canada; ^6^ The Keenan Research Centre for Biomedical Science of St. Michael's Hospital, Unity Health Toronto Toronto Ontario Canada; ^7^ NHC Key Laboratory of Pneumoconiosis, Shanxi Key Laboratory of Respiratory Diseases, Department of Pulmonary and Critical Care Medicine First Hospital of Shanxi Medical University Taiyuan China; ^8^ Health Science Center, Hebei Key Laboratory of Integrated Utilization of Saline Alkali Land in Medical Engineering, Key Laboratory for Quality of Salt Alkali Resistant TCM of Hebei Administration of TCM North China University of Science and Technology Tangshan China

## Abstract

Targeting macrophage SCTR mitigates integrated profibrotic, inflammatory, ER stress, and senescent pathways, preserving lung function and revealing a novel therapeutic strategy for silicosis.

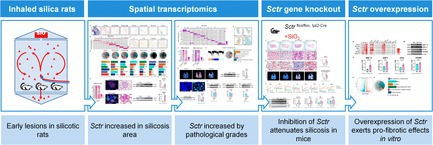

1

Silicosis remains a worldwide occupational health hazard, despite increasing regulation and awareness of dust exposure. In recent years, its emergence in new industries such as hydraulic fracturing, artificial stone fabrication, jewellery polishing, and denim production has resulted in a rise in acute and accelerated cases of the disease [1, 2, 3]. Silicosis is pathologically defined by persistent macrophage‐mediated inflammation, the formation of silicotic nodules, and irreversible pulmonary fibrosis. Despite advances in our understanding of its aetiology, there are currently no effective, approved antifibrotic therapies [4].

Macrophages are centrally involved in the pathogenesis of silicosis, serving as key orchestrators of the inflammatory and fibrotic responses [5]. Their dysregulated activation leads to endothelial cell damage, apoptosis of alveolar epithelial type 1 (AT1) cells [6], disruption of alveolar epithelial type 2 (AT2) cells' homeostasis [7], and transformation of fibroblasts into myofibroblasts [8]. These effects contribute to the accumulation of inflammatory mediators, lipid droplets, and extracellular matrix (ECM), culminating in progressive fibrosis.

Recent advances in spatial transcriptomics have enabled the dissection of heterogeneous tissue architecture with high spatial and transcriptional resolution [9]. This has provided key insights into the cellular and molecular underpinnings of silicosis. Prior studies have identified matrix metalloproteinases‐12 (MMP‐12, *Mmp12*) expressing macrophages as key contributors to silicosis nodule formation and fibrosis [10]. Spatial mapping also reveals complex interactions among macrophage subsets, fibroblasts, and epithelial cells in driving disease progression [11].

Notably, spatial transcriptomics offers the advantage of maintaining tissue context, which is critical in fibrotic lung disease where lesion distribution and microenvironmental factors drive cellular behaviour [12]. By combining histopathology with transcriptional landscapes, this approach reveals not only the identity of cells involved but also their dynamic interactions. In fibrotic regions, macrophage clusters frequently colocalise with fibrotic foci and senescent epithelial populations, suggesting a reciprocal interaction between immune and structural cells [13]. These findings highlight the value of spatial genomics in deciphering the complexity of fibrotic processes and identifying cell‐specific therapeutic targets.

In addition to macrophage‐driven inflammation, increasing evidence suggests that epithelial dysfunction, particularly in AT2 cells, plays a crucial role in silicosis [7]. AT2 cells serve as progenitors for AT1 cells and are critical for alveolar repair. However, under conditions of chronic injury and inflammation, AT2 cells exhibit signs of senescence, characterised by irreversible growth arrest and the secretion of pro‐inflammatory and pro‐fibrotic factors. Senescent AT2 cells lose their regenerative capacity and contribute to the fibrotic milieu [14]. These findings underscore a potential paracrine relationship between activated macrophages and epithelial senescence. In this study, we sought to further elucidate the mechanistic underpinnings of this macrophage–epithelial crosstalk and its contribution to fibrosis.

To explore this further, we developed an inhalation‐cessation rat model of silicosis that mirrors the human disease course following removal from silica exposure [14, 15]. Histopathology confirmed hallmark features, including silica‐laden macrophages, AT2 hyperplasia, AT1 loss, vascular injury, and fibrotic remodelling. Using spatial transcriptomics (300–420 million reads/sample; ∼3000 genes per spot; 2202 spots per tissue section), we defined seven anatomical regions: silicotic core (S), silicotic edge (SE), normal edge (NE), normal parenchyma (N), large vessels (V), bronchi (B), and lymph nodes (L) (Figure 1A, Table S1). Transcriptional profiles correlated well with histologic features, as Kyoto Encyclopedia of Genes and Genomes (KEGG) enrichment analyses consistently matched pathological observations, affirming the reliability of this integrative approach.

**FIGURE 1:**
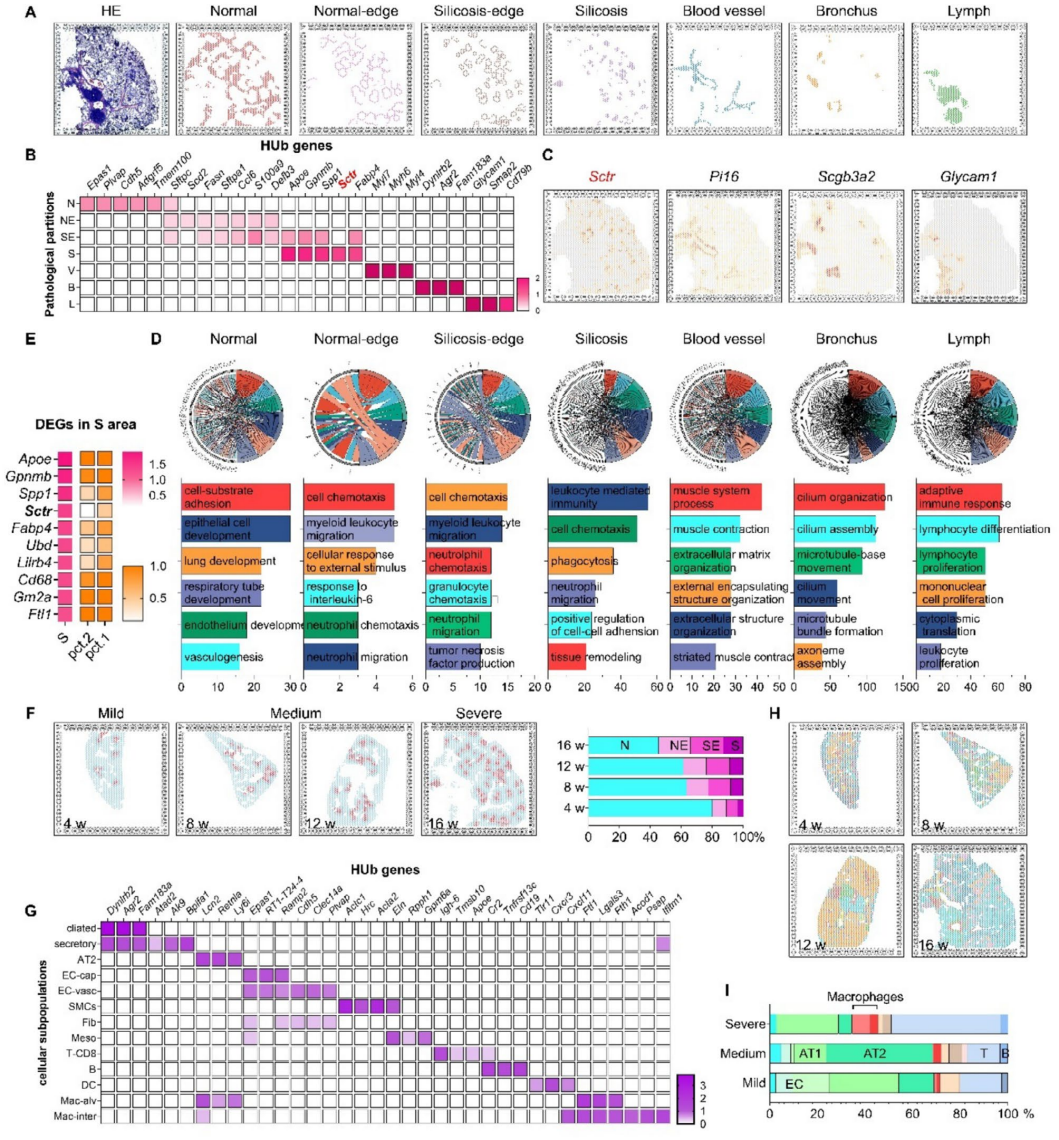
Histological partitions analysed by spatial transcriptomics and cellular subpopulations analysed by spatial transcriptomics. (A) The spatial distribution maps of silicosis (S) area, blood vessel (V), branchus (B), and lymph (L) were classified by the histologic characteristics of silicotic nodules, bronchiole epithelium, smooth muscle, and lymphocyte, respectively. (B) The differentially expressed genes (DEGs) of the pathological partitions. (C) The spatial distribution maps of the selective genes. (D) The GO enrichment diagram of the DEGs of the histological partitions. (E) The DEGs in S areas; S line presents the log2FC of DEGs, PCT.1 represents the proportion of cells expressing a given gene within a specific cell population, whereas PCT.2 represents the proportion of cells expressing that gene within other cell populations. (F) The spatial distribution maps of pathological grades. (G) Cellular subpopulations identified by established lineage markers. (H) The spatial distribution maps of cellular subpopulations in different pathological grades. (I) The rate of cellular subpopulations in different pathological grades.

KEGG analysis revealed enrichment of vascular and epithelial differentiation genes (transmembrane protein 100, *Tmem100* [16]; surfactant protein C, *sftpc*) in N/NE regions, and inflammatory, immune, and matrix‐remodelling pathways in SE/S regions (neutrophil chemotaxis, Interleukin‐1/IL‐6 production, osteoclast differentiation, and collagen biosynthesis) (Figure 1B–D; Table S2). Key macrophage‐related fibrosis genes were among the top hubs in the S area, including phospholipase A2 Group VII (*Pla2g7*), secreted phosphoprotein 1 (*Spp1*), glycoprotein non‐metastatic melanoma protein B (*Gpnmb*), and *Mmp12* [6, 17, 18]. For instance, *Pla2g7* has been shown to promote fibroblast‐to‐myofibroblast transition in Bleomycin‐treated mice via single‐cell RNA‐seq [17]. Similarly, macrophage‐derived *SPP1*, *GPNMB*, and fatty acid binding protein (*FABP5*) have been implicated in scarring and fibrotic foci in both liver and lung fibrosis [18]. MMP‐12, secreted by macrophages, contributes to endothelial cell dysfunction in silicosis by driving tissue injury [6].

Strikingly, the secretin receptor (*Sctr*), a class B1 G‐protein‐coupled receptor (GPCR), emerged as the most significantly enriched hub in the silicotic core (Figure 1E). While SCTR has been implicated in hepatobiliary fibrosis [19, 20], its function in pulmonary disease has not been explored. Silicosis severity was stratified into mild, medium, and severe based on the S‐area proportion (Figure 1F–I). Spatial transcriptomics analysis identified both alveolar macrophage (Mac‐alv) and interstitial macrophage (Mac‐inter) subtypes expressing high *Sctr* levels (Tables [Link], [Link]). *Sctr* expression was highly specific to macrophages and correlated with disease severity. This pattern was validated by immunofluorescence and Western blot (Figure S1A–D). SCTR and its ligand secretin (SCT) were markedly upregulated in silicotic lungs (Figure S1A–D). Pharmacologic blockade of toll‐like receptor 4 (TLR4‐C34) or macrophage depletion via clodronate liposomes significantly reduced SCTR expression (Figure S1E–G), implicating a TLR4‐macrophage axis upstream of SCTR induction.

To evaluate function, we generated macrophage‐specific *Sctr* knockout mice (*Sctr* ^*fl/fl*^; *Lyz2‐Cre* mice). After silica exposure, these mice showed significantly reduced silicotic nodules on histology and micro‐CT (Figures 2A–C and S2A), reduced fibrotic protein levels (pro‐COL I, fibronectin‐1, α‐SMA, SRF) (Figure 2D), and improved lung function (dynamic compliance, airway resistance) (Figures 2F and S2B–D). RNA‐seq of silica‐exposed *Sctr* ^*fl/fl*^ *; Lyz2‐Cre* lungs showed 4269 downregulated transcripts enriched in extracellular matrix (ECM) organisation, transforming growth factor‐β1 (TGF‐β1) signalling, tumour necrosis factor (TNF) signalling, and oxidative‐stress pathways (Figure 2E; Table S7). Conversely, *Sctr overexpressing* in RAW 264.7 macrophages induced 788 transcripts overlapping these same pathways (Figure 2F; Table S8). These findings suggest that SCTR is a central regulator of macrophage‐driven pulmonary fibrosis and a potential therapeutic target for silicosis.

**FIGURE 2:**
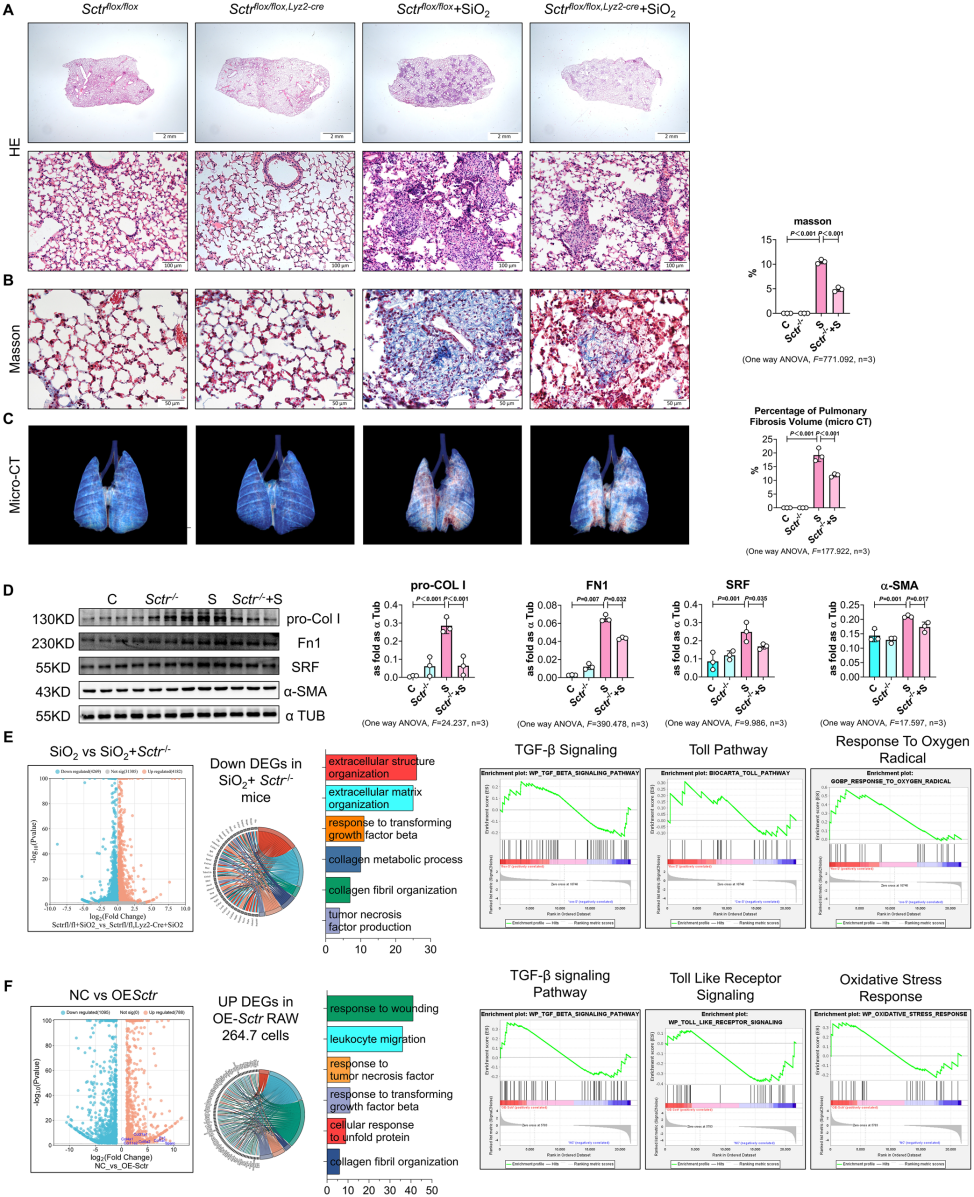
*Sctr* inhibition alleviates collagen deposition in mice exposed to silica. (A, B) The silicotic nodules and the deposition of collagen detected by HE and Masson staining (*n* = 3, scale bars are 2 mm, 100, 50 μm, respectively, one‐way ANOVA, *p* < 0.05). (C) The volume of the high‐density shadow detected by micro‐CT (*n* = 3, one‐way ANOVA, *p* < 0.05). (D) The levels of pro‐Col I, Fn1, SRF, and α‐SMA in *Sctr* ^flox/flox, lyz2‐Cre^ mice exposed to silica measured by western blotting (*n* = 3, one‐way ANOVA, *p* < 0.05). (E) RNA seq of *Sctr*‐overexpressing *Sctr* ^flox/flox, lyz2‐Cre^ mice exposed to silica and the GO and GSEA enrichment of DEGs in *Sctr* ^flox/flox, lyz2‐Cre^ mice exposed to silica. (F) RNA seq of *Sctr*‐overexpressing RAW264.7 cells and the GO and GSEA enrichment of DEGs in *Sctr*‐overexpressing RAW264.7 cells.

Mechanically, overexpression activated canonical TGF‐β1/Smad2/3 signalling in macrophages (Figure S3A,B), and this was attenuated by *Sctr* silencing (Figure S3C,D). Brefeldin‐A confirmed TGF‐β1 secretion (Figure S3E). Cytokine arrays showed that *Sctr*‐overexpressing macrophages also secreted granulocyte colony‐stimulating factor (G‐CSF), CD54, IL‐1β, chemokine (C‐C motif) ligand 3 (CCL3), chemokine (C‐X‐C motif) ligand 12 (CXCL2), and TNF‐α (Figure S3F). These secretions promoted myofibroblast differentiation of mouse lung fibroblasts (MLFs) in co‐culture (elevated COL I, SRF, α‐SMA) (Figure S3G,H), while TGF‐β1, its receptors, and p‐Smad2/3 were downregulated in *Sctr* ^*fl/fl*^ *; Lyz2‐Cre* lungs (Figure S3I).

In parallel, *Sctr* triggered pro‐inflammatory and ER‐stress responses: TLR4, myeloid differentiation primary response protein (MyD88), nuclear factor‐κB (NF‐κB), phosphorylated inhibitor κBα (p‐IκBα), p‐NF‐κB, TNF‐α, IL‐6, and IL‐1β were upregulated by *Sctr* overexpression and suppressed by shRNA knockdown in SiO_2_‐exposed RAW264.7 cells (Figures S4 and S5). ER‐stress markers, including phosphorylated protein kinase r‐like ER kinase (p‐PERK), phosphorylated eukaryotic initiation factor 2 alpha (p‐eIF‐2α), and phosphorylated inositol‐requiring enzyme‐1α (p‐IRE‐1α), were similarly modulated, both in vitro and in *Sctr*‐deficient lungs (Figure S5A,B). The results revealed that macrophage‐specific *Sctr* knockout attenuated silica‐induced fibrosis, as evidenced by reduced collagen deposition and myofibroblast differentiation, impaired TGF‐β1, TLR, TNF‐α, and endoplasmic reticulum stress (ER stress) signalling, and improved lung function.

Given recent evidence that alveolar type II (AT2) cells in silicotic foci undergo senescence via the integrated stress response (ISR) [14], we tested whether SCTR‐positive macrophages modulate this. Spatial transcriptomics showed that differentially expressed genes (DEGs) in AT2 cells were enriched in cell‐cycle arrest and senescence (Figure S6A). Conditioned medium from *Sctr*‐overexpressing macrophages induced phosphorylated ataxia telangiectasia mutated (p‐ATR), phosphorylated ataxia telangiectasia and Rad3‐related protein (p‐ATM), p‐p53^Ser15^, p21, and p16 in MLE‐12 cells (Figure S6B,D). In vivo, β‐galactosidase and pro‐SPC immunostaining confirmed AT2 senescence in silicotic nodules, which were substantially reduced in *Sctr* ^fl/fl^; Lyz2‐Cre lungs (Figure S6E,F). Thus, SCTR‐positive macrophages broadcast pro‐fibrotic and pro‐senescent cues to both mesenchymal and epithelial compartments.

Thess findings reveal that SCTR‐expressing macrophages drive paracrine signalling that exacerbates fibrosis and epithelial senescence. This includes activation of TGF‐β1, TLR4/NF‐κB, and ER‐stress axes that, together, promote myofibroblast differentiation and impair epithelial renewal. Preliminary RNA‐seq hinted at glycolytic reprogramming downstream of SCTR. However, mechanistic data were incomplete and independent of the validated TGF‐β1, TLR4, and ER stress pathways. To avoid speculative interpretation, all glycolysis‐related figures have been removed; this question will be addressed in a dedicated follow‐up study.

In conclusion, our data identify SCTR as a spatially restricted, macrophage‐specific regulator of silica‐induced lung fibrosis. Genetic deletion of *Sctr* in macrophages significantly attenuates fibrosis, inflammation, ER stress, and AT2 cell senescence, and preserves lung function. These findings advance the current understanding of fibrotic lung disease by positioning SCTR at the nexus of macrophage‐driven inflammatory and fibrotic signalling. Importantly, SCTR integrates multiple pathogenic signals—from classical profibrotic cytokines to ER stress and epithelial senescence—suggesting it may serve as a convergent therapeutic target. The availability of small molecule SCTR antagonists developed for liver disease supports the feasibility of pharmacologic intervention [19, 20]. Moving forward, detailed structural and pharmacodynamic studies are warranted to evaluate SCTR inhibition in chronic lung injury models. Furthermore, investigation into human silicosis tissues could determine whether SCTR expression correlates with disease stage or prognosis. As silica exposure remains a threat in both traditional and emerging industries, our findings offer timely insights into the cellular circuits underpinning fibrosis and identify a novel, targetable axis for future therapy.

## Author Contributions

H.X. conceived the project and polished the manuscript. Y.L., T.L, F.J., S.L., and N.M. conducted experiments. W.C., and X.G. performed statistical analysis. H.X., Y.L., Z.W., and D.X. wrote the manuscript and made the figures. H.X., H.Z., Y.S., and F.Y. reviewed the writing and supervised the project.

## Conflicts of Interest

The authors declare no conflicts of interest.

## Supporting information


**Data S1:** Supporting Figures.

**Table S1:** Supporting Tables.

**Table S2:** Supporting Tables.

**Table S3:** Supporting Tables.

**Table S4:** Supporting Tables.

**Table S5:** Supporting Tables.

**Table S6:** Supporting Tables.

**Table S7:** Supporting Tables.

**Table S8:** Supporting Tables.

## Data Availability

The data that support the findings of this study are available from the corresponding author upon reasonable request.
