# Supplementary material for: Targeting the Secretin Receptor in Macrophages Attenuates Silica‐Induced Pulmonary Fibrosis
Source: Cell Prolif. 2025 Sep 18;58(12):e70131. doi: 10.1111/cpr.70131 (PMC12686129; doi:10.1111/cpr.70131)
Supplement: Supplementary file 1 — Data S1: Supporting Figures. [file CPR-58-e70131-s004.docx]

**Supplementary Materials for**

**Targeting the secretin receptor in macrophages attenuates silica-induced pulmonary fibrosis**

Yaqian Li^1#^, Tian Li^2#^, Fuyu Jin^3#^, Shupeng Liu^3^, Dingjie Xu^2^, Zhongqiu Wei^4^, Xuemin Gao^3^, Wenchen Cai^3^, Na Mao^3^, Fang Yang^3^, Haibo Zhang^5,6*^, Yiwei Shi^7*^, Hong Xu^8*^

1. College of TCM, North China University of Science and Technology, Tangshan, China
2. College of Nursing, North China University of Science and Technology, Tangshan, China
3. School of Public Health, North China University of Science and Technology, Tangshan, China
4. Basic Medicine College, North China University of Science and Technology, Tangshan, China
5. Department of Anesthesiology and Pain Medicine, Department of Physiology, Interdepartmental Division of Critical Care Medicine, University of Toronto, Toronto, Ontario, Canada
6. The Keenan Research Centre for Biomedical Science of St. Michael's Hospital, Unity Health Toronto, Ontario, Canada
7. NHC Key Laboratory of Pneumoconiosis, Shanxi Key Laboratory of Respiratory Diseases, Department of Pulmonary and Critical Care Medicine, First Hospital of Shanxi Medical University, Taiyuan, China
8. Health Science Center, Hebei Key Laboratory of Integrated Utilization of Saline alkali Land in Medical Engineering, Key Laboratory for quality of salt alkali resistant TCM of Hebei Administration of TCM, North China University of Science and Technology, Tangshan, China

**MATERIAL AND METHOD**

**1 Establishment of rat and mouse models**

The animals were housed under Specific pathogen-free (SPF) conditions with *ad libitum* food and water in North China University of Science and Technology [SCXK(Ji)2023-018, Tangshan, China]. The study protocol complied with the US National Institutes of Health Guide for the Care and Use of Laboratory Animals and was approved by the ethics committee of North China University of Science and Technology (2024SY3072).

Wistar rats (aged 3 weeks, male) were obtained from the Beijing HFK Bioscience Co., Ltd [SCXK(Jing)2020-0004, Beijing, China]. A HOPE MED 8050 exposure-control apparatus (HOPE Industry and Trade Co. Ltd., Tianjin, China) was used to establish the silicotic rat mode. These rats were exposed to silica for 3 h/day and 5 days/week for a total of 2 weeks. After stopping exposure to silica, the routine feeding was continued, and the samples were collected at 2, 6, 10, and 14 weeks after silica exposure (10 rats at each time point), by adjusting the start time of silica contamination so as to ensure that the samples were collected on the same day and prepared for optimal cutting temperature compound (OCT) embedding and sample delivery. The rats in the control group (n = 10) were placed in the exposure-control apparatus without silica for 3 h/day under the same conditions.

C57BL/6J secretin receptor (*Sctr*) ^[flox/flox,Lyz2-Cre]^ mice (age 8 weeks, half male and half female) were generated by Cyagen Biosciences [SCXK(Su)2022-0016, Suzhou, China]. The silicotic mice model was established via intratracheal instillation of 50 μL of a silica suspension (10 mg/mouse) for 4 weeks; the control rats were treated with 0.9% saline. For macrophage depletion, the treatment method was as described in our previous study^1^.

**2 Cell culture**

RAW264.7, MLE-12, MLF, and 293T cell lines were obtained from National Collection Authenticated Cell Cultures (China). RAW264.7 was cultured in Ham’s F12K medium supplemented with 20% fetal bovine serum (FBS); MLE-12 was cultured in DMEM/F12 medium supplemented with 10% BFS; MLF was cultured in DMEM medium supplemented with 10% FBS; 293T was cultured in DMEM medium supplemented with 10% FBS; all cells were cultured in a humidified atmosphere with 5% CO_2_ at 37°C.

RAW 264.7 murine macrophage cell lines were treated without or with one or more of the following: 50 µg/mL silica (Sigma-Aldrich) or treated without or with Toll-like receptor 4 (TLR4)-C34 (18512, Cayman Chemical).

The *Sctr* CRISPR activation plasmid (sc-435601-ACT, Santa Cruz) or *Sctr* shRNA plasmid (sc-153320-SH, Santa Cruz) was transfected into 293T cells using lip2000 transfection reagent (s5631, Thermo Fisher Scientific), and the lentiviral particles were collected at 48–72 h after transfection. The RAW264.7 cells were infected with lentivirus particles and selected by puromycin (13884, Cayman Chemical) to obtain a stable cell line. Western blotting was performed to detect the levels of target proteins in the stable cell lines.

**3 Co-culture of OE-*Sctr* RAW264.7 cells and MLF.MLE-12 cells**

RAW264.7 cells overexpressing *Sctr* were plated in the bottom chamber of Tissue Culture Plate Insert (TCS-016-006, Guangzhou Jet Bio-Filtration). After full adherence was achieved, MLF/MLE-12 cells were seeded in the top chamber. Following a 24-hour incubation period, proteins were extracted from the MLF cells for downstream experiments.

**4 Spatial transcriptomics**

The lung tissues were sliced into small pieces of approximately 0.8 cm in length, width, and height. The surface liquid was dried with an absorbent paper. The lung tissue was then immersed in pre-cooled isopentane for 1 min and then placed in an embedding mold that had been partially filled with OCT. OCT was continuously added to the mold to completely cover the lung tissues, avoiding air bubble generation in and around the tissues. Once the OCT was completely frozen, it was stored at -80°C for sample submission.

Space transcriptome sequencing was conducted by Beijing Geek Technology Co., Ltd. Paired-ended sequencing using Illumina was performed for sequencing, and the library construction method used was 10X Genomics. After passing the quality inspection, Space Ranger was used to generating a function barcode matrix as input data, through Seurat, and a series of programs were performed as standard preprocessing on the data. 'sctransform' was used to normalize the data and detect high variable feature genes. tSNE was used to reduce the multidimensional PC image to a 2-dimensional image so as to demonstrate the distribution of cell expressions.

A harmony package was used to integrate the sequencing data of 4 samples. Differential gene screening and gene enrichment analysis were performed, and all differential gene screening criteria were Log_2_FC>0.25 and *P* < 0.05. The selected differential genes were subjected to Gene Ontology (GO) and the Kyoto Encyclopedia of Genes and Genomes (KEGG). ClusterProfiler package, pathview package, GOplot package, and ggpubr package were used for the KEGG pathway enrichment analysis and plotted.

**5 Micro CT imaging**

High-resolution CT images of mouse lungs were obtained by using the NEMO-II NMC-200 machine (Pingsheng, Jiangsu, China) in accordance with the manufacturer's protocol. The mice were in the prone position. During the shooting, ensure that the animal cabin door was closed and the baffle was returned to its original position. Mice were anesthetized with isoflurane and the micro-CT scanning mode (tube voltage 60 kV, current 130 µA, resolution 1K x 1K, pixel size 0.050 mm, slice thickness 0.050 mm, scan time 5 min) was set. Subsequently, the acquired images were processed using the AVATAR 1.5.0 software to generate three-dimensional representations of the lung tissues.

**6 Pulmonary function measurement**

The FinePointe WBP system (Buxco Research Systems, Wilmington, NC, USA) was employed to gauge the pulmonary functions in conscious mice while strictly adhering to the manufacturer's instructions. The pulmonary function indices were auto-detected at 2-s intervals. The protocol involved an adaptation period of 8 min, followed by a nebulization phase lasting 1 s, a response period of 5 min, and a recovery duration of 1 min. The key indices examined included tidal volume (Tvb), minute volume (Mvb), expiratory flow 50 (EF50), peak expiratory flow (PEF), peak inspiratory flow (PIF), and inspiratory time (Ti).

**7 High-throughput transcriptome sequencing**

High-throughput transcriptome sequencing services were provided by Guangzhou Ribo Bio Co., Ltd (Guangzhou, China). Lung tissue samples were collected from each group of mice and immediately frozen in liquid nitrogen. After RNA extraction, purification, and library establishment, the libraries were paired-end sequenced using next-generation sequencing based on the Illumina sequencing platform.

**8 H&E staining, Masson staining and SA-β-gal staining**

Paraffin-embedded lung tissue sections were subjected to deparaffinization and rehydration, followed by the relevant staining protocols.

**9 Immunofluorescence (IF) staining**

The sections were incubated with SCTR (sc-166112, B2410, Santa Cruz), Cluster of Differentiation 68 (CD68, ab201340, 1008461-2, Abcam), pro-collage I (COL I, ab34710, Abcam), α-smooth muscle actin (α-SMA, ET1607-53, HUABIO), Toll-like receptor 4 (TLR4, ARG20515, arigo), transforming growth factor-β1 (TGF-β1, ARG56429, 100709, arigo), phosphorylated protein kinase r-like ER kinase (p-PERK) (DF7576, 3k58441, Affinity) and β-galactosidase (HA500021, HN0724, HUABIO), respectively, and then combined with the donkey anti-mouse tetraethyl rhodamine isothiocyanate and donkey anti-rabbit fluorescein isothiocyanate isomer I secondary antibodies (074-1506/074-1806, Kirkegaard and Perry Laboratories).

**10 Western blotting**

The process of protein separation was conducted via SDS-PAGE and subsequently detected using western blotting, as delineated in previous work ^2^. Briefly, the membranes underwent treatment with primary antibodies first for Secretin (SCT, GTX129460, GeneTex) COL I, α-SMA, TLR4, Myeloid differentiation primary response protein (MyD88, ab2068, GR102267-3, Abcam), Nuclear factor kappa-B (NF-κB, ARG51013, arigo), p-NF-κB (ARG51516, arigo), phosphorylated inhibitor I kappa-B α (p-IκBα, AF2002, Affinity), tumor necrosis factor-α (TNF α, GTX110520, 43614, GeneTex), Interleukin-6 (IL-6, A0286, Abclonal), IL-1β (DF6251, Affinity), TGF-β1, TGF-β-RII (ARG59501, 80911, arigo), p-Smad2/3 (ARG40897, arigo), Smad2/3 (ARG43376, arigo), PERK (HA721510, HUABIO), phosphorylated eukaryotic initiation factor 2 alpha (p-eIF-2α, ET1603-14, HM0708, HUABIO), eIF-2α (ET7111-34, HN0810, HUABIO), phosphorylated inositol-requiring enzyme-1α (p-IRE-1α, ab48187, Abcam), IRE-1α (A00683-1, Boster), phosphorylated ataxia telangiectasia and Rad3-related protein (p-ATR, DF7512, Affinity), ATR (A00262, Boster), phosphorylated ataxia telangiectasia mutated (p-ATM, AF8410, Affinity), ATM (ARG23515, arigo), p-p53-S15 (AP0083, Abclonal), p53 (ARG10519, arigo), p21 (ab109520, GR3192685-6, Abcam), p16 (A0262, Abclonal), β-actin (R13025, HUABIO), and α-TUB (HA721271, HUABIO), each diluted into a 1:1000 ratio. Thereafter, the membranes were subjected to incubation with goat anti-rabbit or goat anti-mouse secondary antibodies (1:5000 dilution) within a blocking buffer medium. The immunoblots were subsequently visualized using an ECL prime Western blotting detection reagent, with results normalized against the corresponding controls.

**11 Coomassie blue staining**

Remove the polyacrylamide gel after electrophoresis and place it into a plastic container. Add an appropriate amount of rapid staining solution (B822606, RW0411, Rrport Biotech) to cover and submerge the gel. After 8 hours, discard the staining solution, rinse the gel with pure water for 40 minutes, and then capture the image.

**12 Proteome Profiler Array**

Collect the cell culture medium from RAW264.7 cells either silica-induced for 48 hours or overexpressing *Sctr*. After filtering the medium through a 0.22-μm filter, incubate it with the nitrocellulose membranes each containing 40 different capture antibodies for at least 8 hours according to the instructions of the Proteome Profiler kit (ARY006, P346170). Subsequently, wash the membrane, incubate with Streptavidin-HRP for 30 minutes, wash again, and finally detect signals using chemiluminescence after adding the substrate solution.

**13 Statistical Analysis**

Statistical analyses were performed in R version 4.2.2 or SPSS v23.0, unless otherwise indicated. No data were excluded from the studies and for all experiments, and all attempts at replication were successful. For each experiment, the sample size reflects the number of independent biological replicates, which is provided in the figure legend. Statistical analyses of single comparisons of two groups employed Student’s *t*-test. For multiple comparisons, one-way analysis of variance (ANOVA) was employed, followed by host-hoc analysis using Tukey’s test. Statistical significance was considered achieved at *p* < 0.05, within a 95% confidence interval. The results were considered to be significant at *p* < 0.05, where **p* < 0.05. Statistical details are provided in the respective figure legends.

**Supplementary Figures**


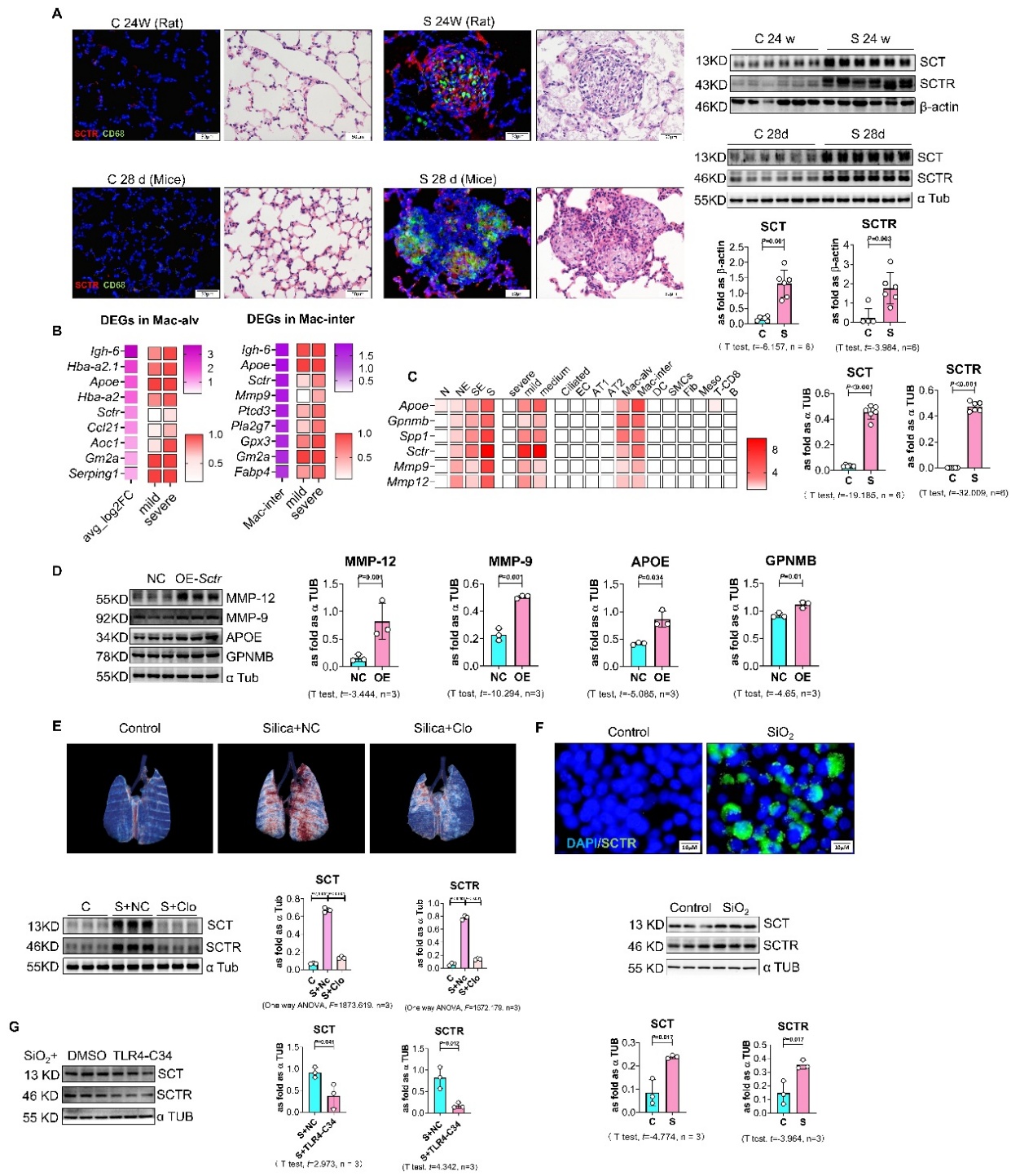


**Supplementary Figure 1** (A) the co-expression of CD68 and SCTR in silicotic rodents were detected by immunofluorescence (IF) staining. (scale bars are 50 μm). The level of SCT and SCTR in siliotic rodents detected by western blot (n=6, T test, *P* < 0.05). (B) The expression level and cellular specificity of DEGs in alveolar macrophage (Mac-alv) and in interstitial macrophages (Mac-inter). (C) The levels of *Sctr* and co-expressed DEGs, including *Apoe*, *Gpnmb*, *Mmp9*, and *Mmp12* in pathological partitions, pathological grades, and cellular subpopulations analyzed by spatial transcriptomics. (D) The levels of APOE, GPNMB, MMP9, and MMP12 in *Sctr*-overexpressing RAW 264.7 cells detected by and western blot (n=3, T test, *P*< 0.05). (E) The volume of the high-density shadow detected by micro-CT (n=3, one-way ANOVA, *P* < 0.05); The level of SCT and SCTR measured by Western blot in silicotic mice by depletion of macrophages (n=3, one-way ANOVA, *P*< 0.05). (F) The expression of SCTR and the level of SCT and SCTR in silica-induced RAW 264.7 cells detected by IF staining (n=3, T test, *P* < 0.05) and Western blot (n=3, T test, *P* < 0.05). (G) The level of SCT and SCTR measured by Western blot in silica-treater RAW 264.7 cells treatment with TLR4-C34 (n=3, T test, *P* < 0.05).

**
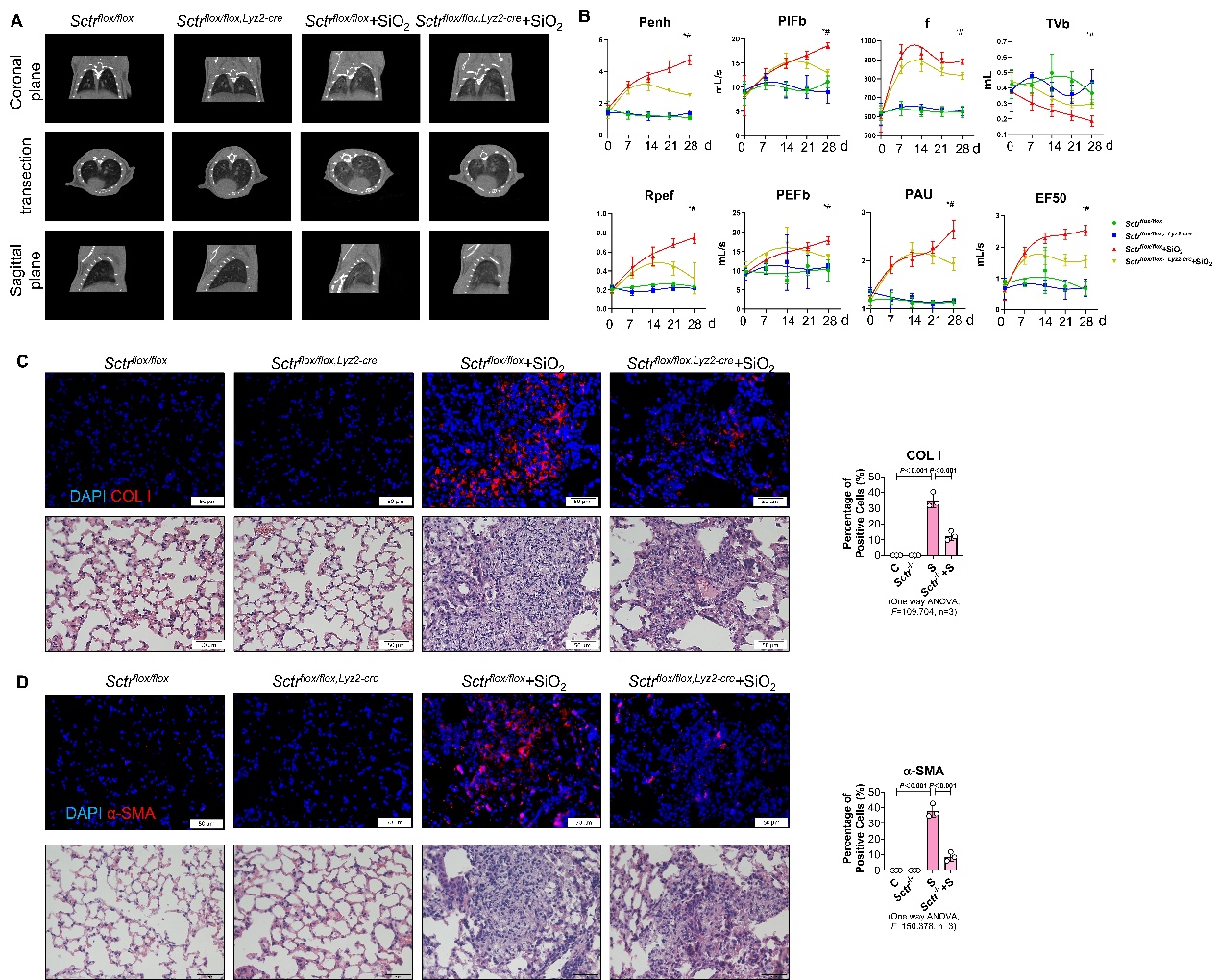
**

**Supplementary Figure 2** (A) micro-CT of silicotic mice. (B) Lung functions of silicotic mice. (C) The positive expression of COL I in silicotic nodules observed by IF staining (n=3, scale bars are 50 μm, respectively, one-way ANOVA, *P* < 0.05). (D) The positive expression of α-SMA in silicotic nodules observed by IF staining (n=3, scale bars are 50 μm, respectively, one-way ANOVA, *P* < 0.05).


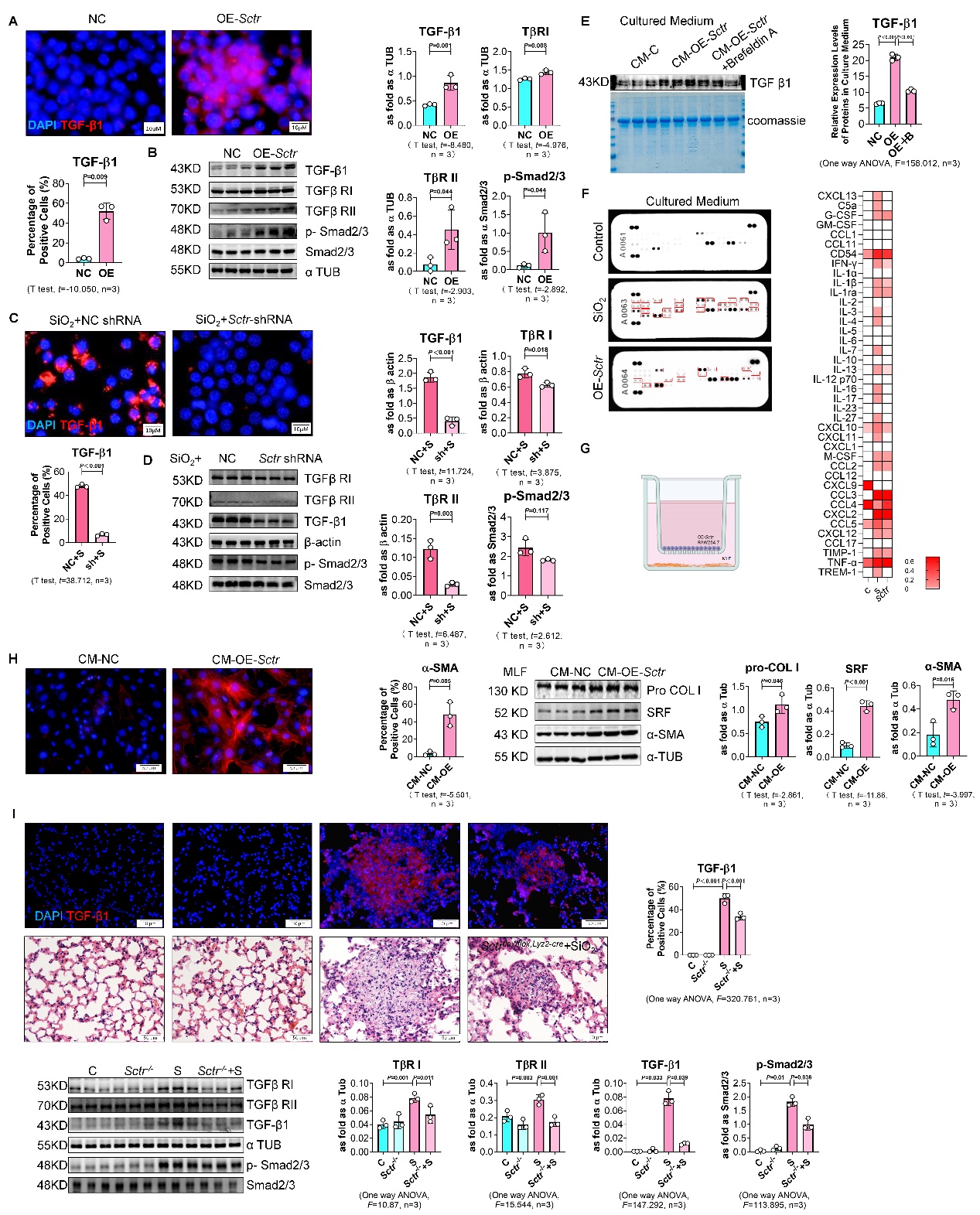


**Supplementary Figure 3** (A) The positive expression of TGF-β1 in *Sctr-*overexpressing RAW 264.7 cells observed by IF staining (n=3, scale bars are 10 μm, T test, *P* < 0.05). (B) The levels of TGF-β1 pathway related protein in *Sctr-*overexpressing RAW 264.7 cells detected by and western blot (n=3, T test, *P*< 0.05). (C) The positive expression of TGF-β1 in silica-induced RAW 264.7 cells treatment with *Sctr* shRNA observed by IF staining (n=3, scale bars are 10 μm, T test, *P* < 0.05). (D) The levels of TGF-β1 pathway related protein in silica-induced RAW 264.7 cells treatment with *Sctr* shRNA measured by western blot (n=3, T test, *P* < 0.05). (E) The level of TGF-β1 measure by Western blot in conditional medium derived from NC/ *Sctr*-overexpressing/ *Sctr*-overexpressing +Brefeldin A RAW 264.7 cells (n=3, T test, *P* < 0.05). (F) Mouse Cytokine Array of the medium of *Sctr-*overexpressing or silica-treated RAW 264.7 cells. G) Co-cultured model of RAW 264.7 and MLF; H) the positive expression of α-SMA in MLF co-cultured with *Sctr-*overexpressing RAW 264.7 cells measured by IF staining (n=3, scale bars are 10 μm, T test, *P* < 0.05); The levels of pro-Col I, SRF, and α-SMA in MLF co-cultured with *Sctr-*overexpressing RAW 264.7 cells measured by Western blot (n=3, T test, *P* < 0.05). (H) The positive expression of TGF-β1 in silicotic nodules observed by IF staining (n=3, scale bars are 50 μm, respectively, one-way ANOVA, *P* < 0.05). (I) The levels of TGF-β1 signaling pathway related proteins in *Sctr* ^flox/flox, lyz2-Cre^ mcie exposed to silica measured by Western blot (n=3, one-way ANOVA, *P* < 0.05).


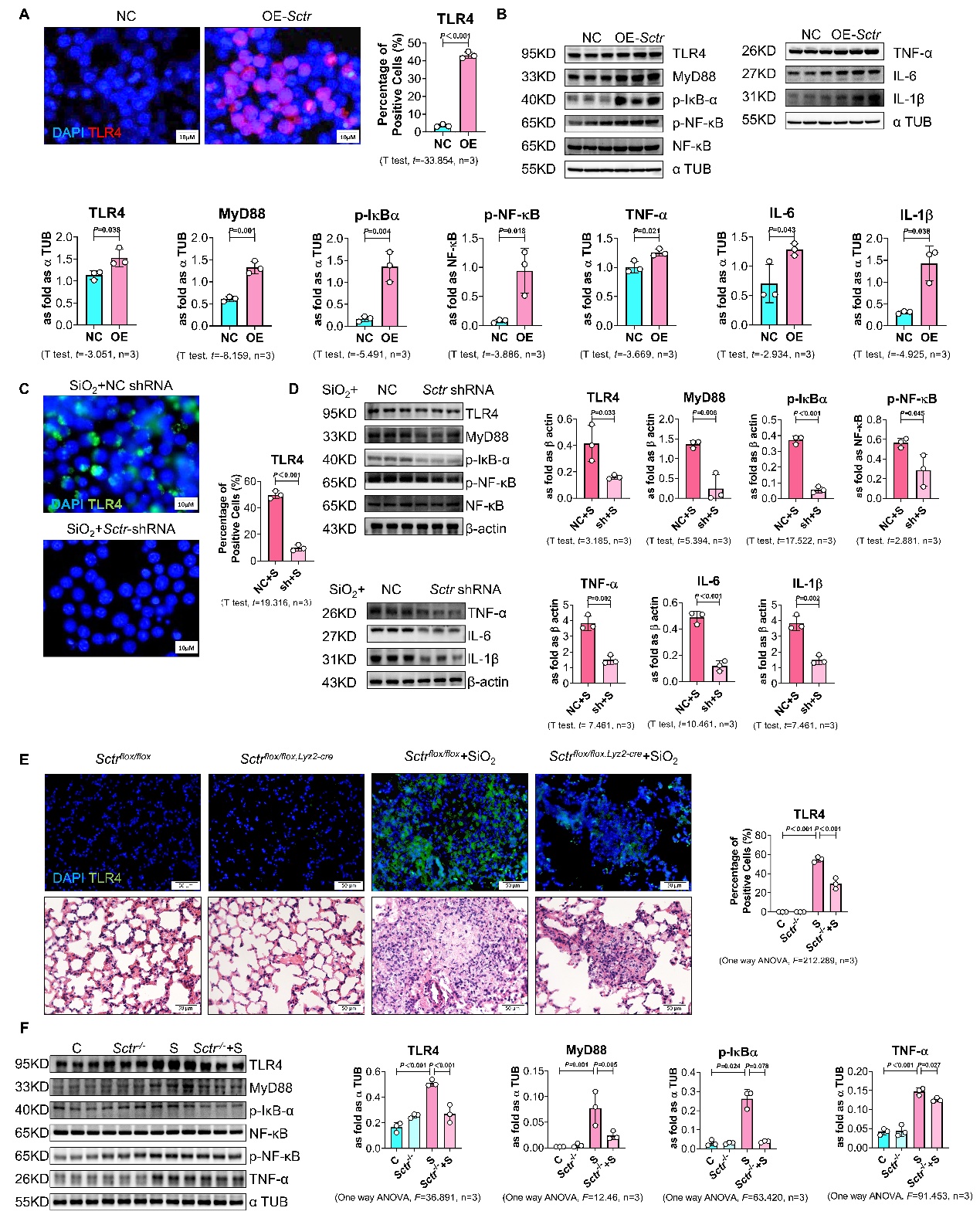


**Supplementary Figure 4** (A) The positive expression of TLR4 observed by IF staining in *Sctr*-overexpressing RAW 264.7 cells (n=3, scale bars are 10 μm, T test, *P* < 0.05). (B) The levels of TLR4, MyD88, p-IκBα, p-NF-κB, TNF-α, IL-6, and IL-1β in *Sctr-*overexpressing RAW 264.7 cells measured by Western blot (n=3, T test, *P* < 0.05). (C) The positive expression of TLR4 observed by IF staining in silica-induced RAW 264.7 cells treatment with *Sctr* shRNA (n=3, scale bars are 10 μm, T test, *P* < 0.05). (D) The levels of TLR4, MyD88, p-IκBα, p-NF-κB, TNF-α, IL-6, and IL-1β in silica-induced RAW 264.7 cells treatment with *Sctr* shRNA measured by Western blot (n=3, T test, *P* < 0.05). (E) The positive expression of TLR4 in silicotic nodules observed by IF staining (n=3, scale bars are 50 μm, respectively, one-way ANOVA, *P* < 0.05). (F) The levels of TLR4 signaling pathway related proteins in *Sctr* ^flox/flox, lyz2-Cre^ mcie exposed to silica measured by Western blot (n=3, one-way ANOVA, *P* < 0.05).


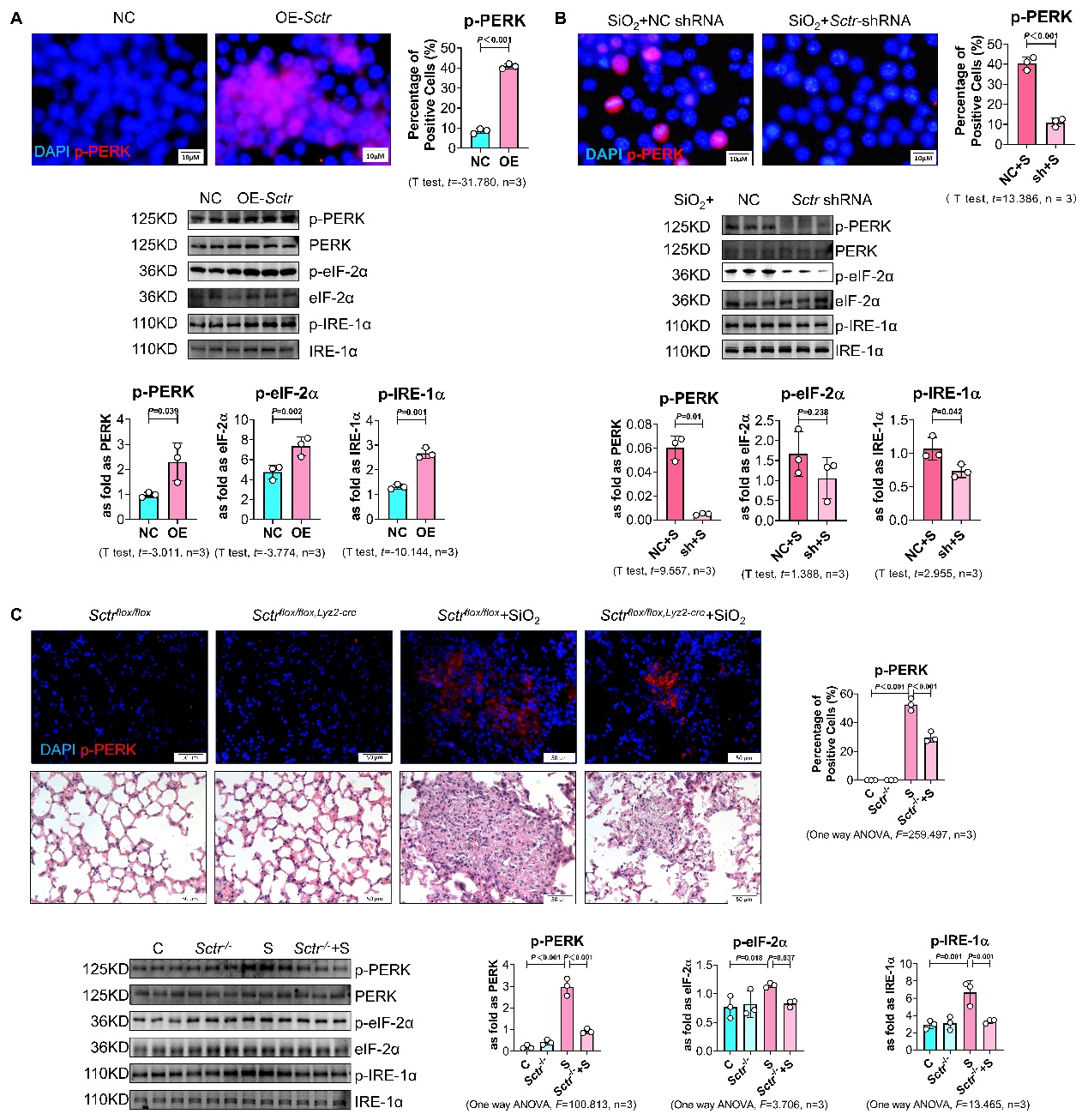


**Supplementary Figure 5** (A) Positive expression of p-PERK in *Sctr*-overexpressing RAW 264.7 cells observed by IF staining (n=3, scale bars are 10 μm, T test, *P* < 0.05). The levels of p-PERK, p-eIF-2α, and p-IRE-1α in *Sctr*-overexpressing RAW 264.7 cells measured by Western blot (n=3, T test, *P* < 0.05). (B) Positive expression of p-PERK in silica-induced RAW 264.7 cells treatment with *Sctr* siRNA observed by IF staining (n=3, scale bars are 10 μm, T test, *P* < 0.05). The levels of p-PERK, p-eIF-2α, and p-IRE-1α in silica-induced RAW 264.7 cells treatment with *Sctr* siRNA measured by Western blot (n=3, T test, *P* < 0.05). (C) The levels of ER stress related proteins in *Sctr* ^flox/flox, lyz2-Cre^ mcie exposed to silica measured by Western blot (n=3, one-way ANOVA, *P* < 0.05).


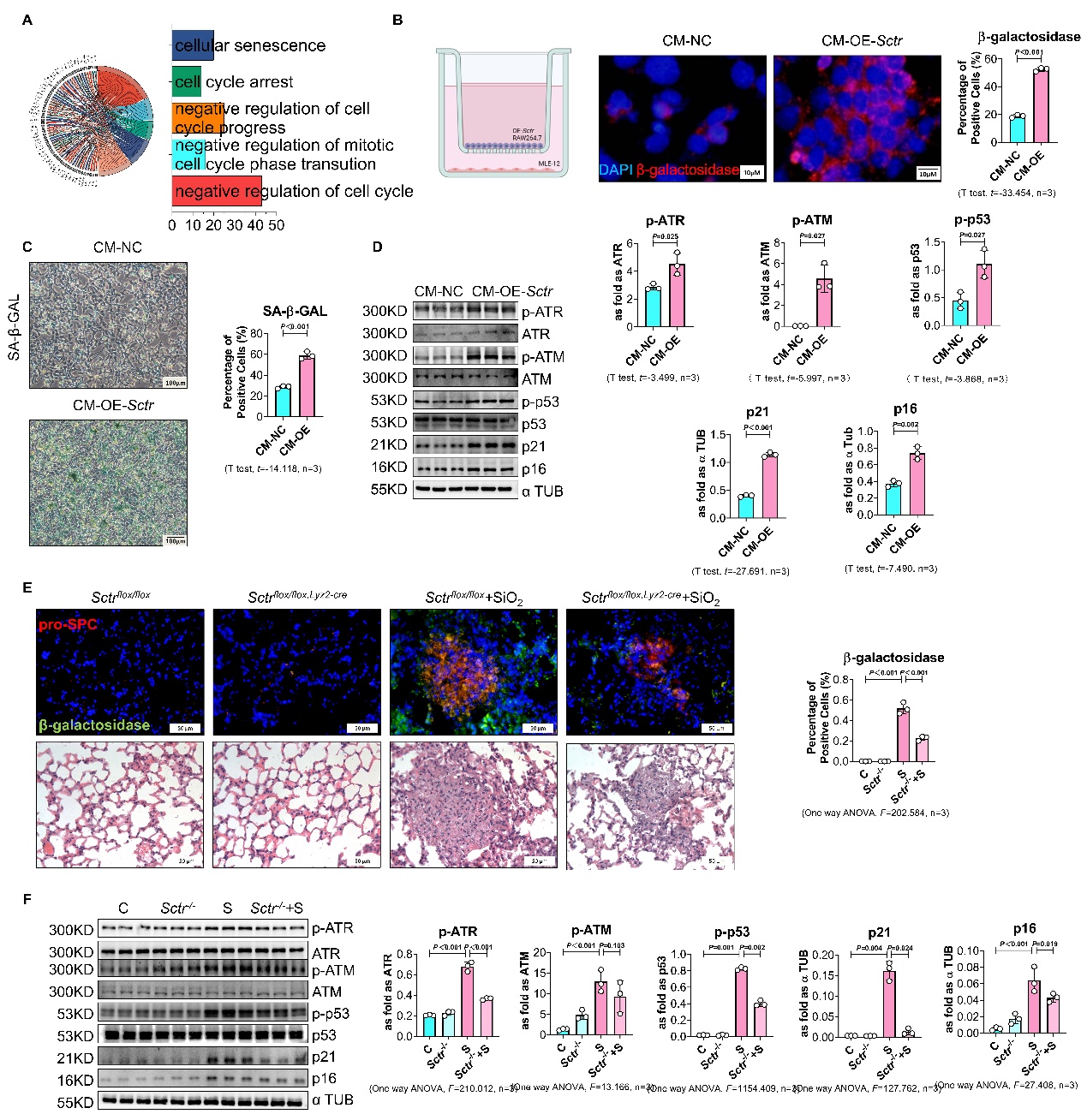

**Supplementary Figure 6** (A) GO analysis of DEGs in AT2 cells. (B-C) Co-cultured model of RAW 264.7 and MLE-12 cells; the positive expression of β-galactosidase (scale bars are 10 μm) and SA-β-GAL staining (scale bars are 100 μm) in MLE-12 cells co-cultured with *Sctr*-overexpressing RAW 264.7 cells (n=3, T test, *P* < 0.05). (D) The levels of cellular senescence related proteins in MLE-12 cells co-cultured with *Sctr*-overexpressing RAW 264.7 cells measured by Western blot (n=3, T test, *P* < 0.05). (E) The positive expression of pro-SP C and β-galactosidase in silicotic nodules observed by IF staining (n=3, scale bars are 50 μm, respectively, one-way ANOVA, *P* < 0.05) (F) The levels of cellular senescence related proteins in Sctr flox/flox, lyz2-Cre mcie exposed to silica measured by Western blot (n=3, one-way ANOVA, *P* < 0.05).

1. Jin F, Li Y, Gao X, et al. Exercise training inhibits macrophage-derived IL-17A-CXCL5-CXCR2 inflammatory axis to attenuate pulmonary fibrosis in mice exposed to silica. *Sci Total Environ.* 2023;902:166443.

2. Li T, Mao N, Xie Z, et al. Paeoniflorin mitigates MMP-12 inflammation in silicosis via Yang-Yin-Qing-Fei Decoction in murine models. *Phytomedicine.* 2024;129:155616.
